# Characterization of Nanoparticle Intestinal Transport Using an In Vitro Co-Culture Model

**DOI:** 10.3390/nano9010005

**Published:** 2018-12-21

**Authors:** Alina F.G. Strugari, Miruna S. Stan, Sami Gharbia, Anca Hermenean, Anca Dinischiotu

**Affiliations:** 1 University of Bucharest, Faculty of Biology, Department of Biochemistry and Molecular Biology, 91-95 Splaiul Independentei, 050095 Bucharest, Romania; alina.strugari@gmail.com (A.F.G.S.); ancadinischiotu@yahoo.com (A.D.); 2 Institute of Life Sciences, Vasile Goldis Western University of Arad, 86 Rebreanu, 310414 Arad, Romania; samithgh2@hotmail.com (S.G.); anca.hermenean@gmail.com (A.H.); 3 Department of Histology, Faculty of Medicine, Pharmacy and Dentistry, Vasile Goldis Western University of Arad, 1 Feleacului, 310396 Arad, Romania

**Keywords:** co-culture intestinal model, Caco-2, HT29-MTX, nanoparticle transport, quantum dots, iron oxide nanoparticles

## Abstract

We aimed to obtain a tunable intestinal model and study the transport of different types of nanoparticles. Caco-2/HT29-MTX co-cultures of different seeding ratios (7:3 and 5:5), cultured on Transwell® systems, were exposed to non-cytotoxic concentration levels (20 μg/mL) of silicon quantum dots and iron oxide (α-Fe_2_O_3_) nanoparticles. Transepithelial electric resistance was measured before and after exposure, and permeability was assessed via the paracellular marker Lucifer Yellow. At regular intervals during the 3 h transport study, samples were collected from the basolateral compartments for the detection and quantitative testing of nanoparticles. Cell morphology characterization was done using phalloidin-FITC/DAPI labeling, and Alcian Blue/eosin staining was performed on insert cross-sections in order to compare the intestinal models and evaluate the production of mucins. Morphological alterations of the Caco-2/HT29-MTX (7:3 ratio) co-cultures were observed at the end of the transport study compared with the controls. The nanoparticle suspensions tested did not diffuse across the intestinal model and were not detected in the receiving compartments, probably due to their tendency to precipitate at the monolayer surface level and form visible aggregates. These preliminary results indicate the need for further nanoparticle functionalization in order to appropriately assess intestinal absorption in vitro.

## 1. Introduction

Oral drug administration is the preferred route when it comes to delivering most active compounds, with advantages including patient comfort, reduced chances of infection, and minimal invasiveness compared with alternative delivery systems. The assimilation process via the gastrointestinal (GI) tract is a paramount condition for the delivery of any xenobiotic active compound to target tissues. In order to reach the vascular circulation, orally administered drugs usually have to pass through the small intestinal barrier. However, some active compounds are especially vulnerable to the harsh GI environment and, therefore, require protection during transit in order to prevent degradation [1]. In this context, nanoparticles constitute novel candidates as future carrier-type agents [2]. Furthermore, human exposure to food products containing nanoparticulate materials is projected to increase in the near future, which calls for the development of a reliable screening solution [3].

The intestinal mucosa constitutes the major absorption site in vivo, with nutrient and xenobiotics having to penetrate two types of barriers—an acellular mucus layer and the *intestinal epithelium*. The latter is composed of a heterogeneous population of terminally differentiated cells which are constantly renewed by Lgr5+ and +4 stem cells located at the crypt base level, making the mammalian intestinal epithelium renowned for having one of the highest turnover rates in the body [4]. Up to 80% of the cells that make up the tissue are absorptive enterocytes, followed by mucus-secreting goblet cells which, ratio-wise, vary between 10 and 24%, depending on the GI tract region in which they are located [5]. Lymphatic follicles cluster in different regions of the ileum and are known as Peyer’s patches. These patches are covered by a specialized tissue called the follicle-associated epithelium (FAE), which is composed of mainly enterocytes and M (microfold) cells that interact with the local immune system, mediating antigenic influx as well as bacterial attachment [2,6,7]. Paneth immune cells are also part of the epithelium—located at the base of Lieberkühn’s crypts, they produce acidophilic granules containing antibacterial and digestive enzymes. Enteroendocrine cells account for around 1% of the intestinal monolayer and are sporadically dispersed throughout the intestinal mucosa [4]. 

**Figure 1:**
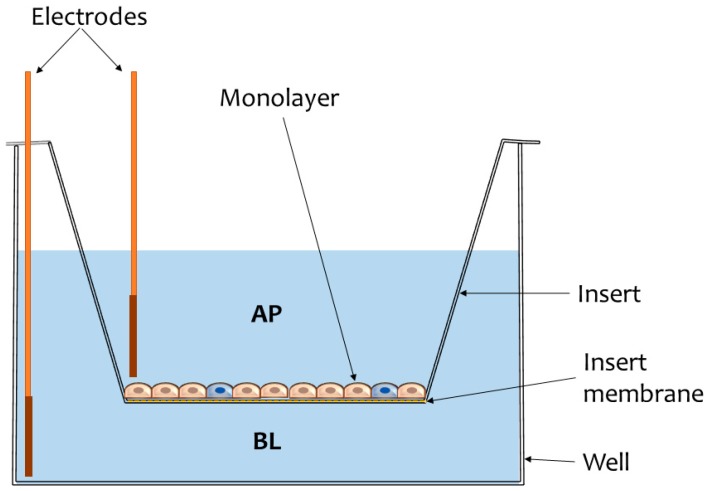
Illustration of a common experimental model used in intestinal transport studies. The cells are cultivated on inserts supplied with semipermeable membranes through which microparticulate diffusion may occur. Monolayer integrity is usually assessed by measuring transepithelial electrical resistance (TEER) using chopstick electrodes inserted in the apical (AP) and basolateral (BL) compartments of the well.

There is currently a wide array of in vitro intestinal models being used in studies that aim to estimate/predict drug absorption in vivo. Even though there has been a surge in the development of organotypic (3D) models [8,9,10] and organs-on-chip (body-on-a-chip) systems [11,12], the majority of transport studies rely on simpler, in vitro co-culture models using conventional cell lines. There are many advantages in terms of cost and their good reproducibility and fidelity, yet the use of tumoral cell lines (which is common practice in most 2D in vitro intestinal models) raises several concerns regarding the ability of the models to accurately reflect in vivo intestinal absorption. More often than not, tumoral cells are found to overexpress key proteins [13,14], and they generally exhibit an altered transcriptional regulation phenotype that may impact tissue permeability. For example, the Caco-2 adenocarcinoma cell line has been used extensively for the past couple of decades in nutrient and drug transport studies as an adequate in vitro model of the intestinal mucosa [15,16]. However, due to the overexpression of tight junction protein complexes [5,17,18,19,20], simple Caco-2 monolayers fail to provide a reliable estimation of in vivo paracellular permeability of small hydrophilic compounds. To address this issue, Caco-2 cells are routinely co-cultured alongside HT29-MTX (goblet-like) cells [18] on Transwell^®^ inserts (Figure 1).

This co-culture model allows for modulating intercellular junction geometry, thereby fine-tuning the *effective permeability* (*P*_eff_) of the monolayer by simply adjusting the initial cell seeding ratio [17]. The human adenocarcinoma line HT-29 preconditioned in methotrexate (MTX) has the added benefit of expressing mucins in culture [21]—the resultant mucus layer produced constitutes an additional physical barrier [6], potentially impeding xenobiotic transport across the epithelium as would be the case under in vivo conditions. Intestinal permeability correlates with the rate of compound transport across the mucosa, which is calculated according to the following equation:

(1) $$P_{\mathrm{eff}}=\frac{dQ\;V}{dt\;A\;C_{0}}\;\left[\mathrm{cm}/s\right],$$

where *dQ/dt* represents the apparent flow rate in time across the monolayer (mM/mL∙s), *V* is the volume (mL) within the receiving compartment (BL), *C*_0_ is the initial concentration of the compound (mM) in the donor compartment (AP), and *A* is the exposed tissue surface area (cm^2^). 

**Figure 2:**
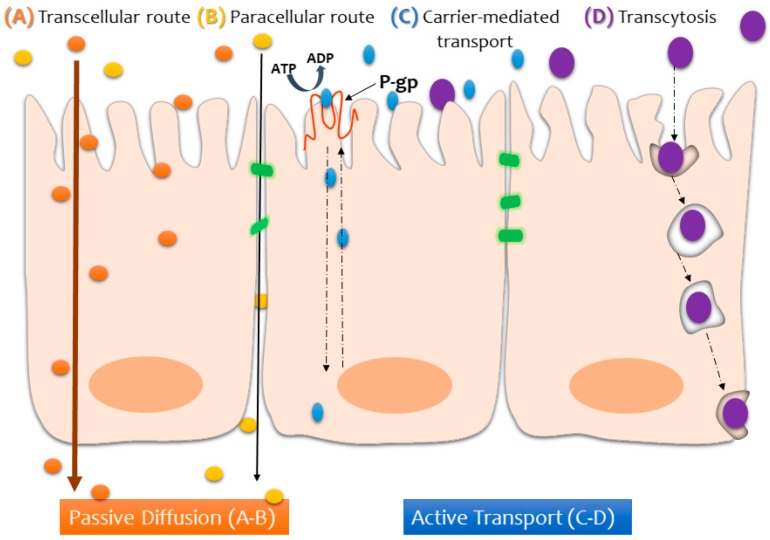
Schematic illustration of the possible transport pathways across the intestinal epithelium, depending on the nature of the compound being absorbed.

The physicochemical properties of the compounds that are tested in transport studies also have a huge impact on the rate of absorption and, in some cases, require functionalization in order to facilitate (or even allow) assimilation across the intestinal mucosa [3]. Depending on the nature of the compound, it may reach the other side of the epithelium by way of the transcellular or paracellular route (via passive diffusion), transcytosis, or active transport (via carrier-mediated transport), as depicted in Figure 2.

It is worth keeping in mind that, in reality, xenobiotic assimilation depends on many more variables. Bioavailability (*F*) is among the most crucial parameters in the field of pharmacokinetics, as it describes the fraction of compounds that manage to penetrate the intestinal barrier and reach blood circulation in their unaltered form [22]:

*F* = *f_a_* (1 − *E*_G_) ∙ (1 − *E*_H_), (2)

where *f_a_* is the absorbed fraction of the dose administrated (mass/dose), while considering first-pass metabolism of the compound in the gut wall (*E*_G_) and liver (*E*_H_). These are variables which current in vitro co-culture models cannot account for.

Although Caco-2/HT29-MTX co-cultures are routinely used in transport studies, the model is yet to be fully characterized, especially when compared with the well-established Caco-2 monoculture model. The present study aims to add to the already established body of work in this direction [5,23] while assessing the potential of two types of nanoparticles for oral drug delivery or screening perspectives. We established several Caco-2/HT29-MTX cultures by altering the initial seeding ratios (10:0, 7:3, 5:5, 0:10). The cells were cultured using Transwell^®^ systems and were allowed to develop into stable monolayers for 21 days before exposing them to non-cytotoxic concentrations (20 μg/mL) of silicon quantum dots (Si QDs) and iron oxide (α-Fe_2_O_3_) nanoparticles. According to our previous investigations [24,25], we chose to conduct the transport study using the concentration of 20 μg/mL for both types of nanoparticles. Those particular studies showed that this dose does not induce changes in the number of viable cells or have cytotoxic effects. Using a lower concentration would not be appropriate for the objective of the study, as it could impede proper particle detection by conventional methods and alter the particles’ potential drug delivery function, while a higher concentration results in more aggregates and increased cell toxicity. Transepithelial electric resistance (TEER) was measured before and after exposure, and monolayer permeability (*P*_eff_) was assessed via the paracellular marker Lucifer Yellow. At regular intervals during the 3-h transport study, samples were collected from the basolateral compartments for detection and quantitative testing. Cell morphology characterization was done by actin cytoskeleton labeling, and Alcian Blue/eosin staining was performed on insert cross-sections in order to compare the intestinal models and evaluate the production of mucins.

## 2. Materials and Methods 

### 2.1. Nanoparticles

The Si QDs and α-Fe_2_O_3_ nanoparticles used in this study were produced at the Laser Department from the National Institute of Lasers, Plasma and Radiation Physics, Bucharest-Măgurele. The silicon nanoparticles were synthesized via a pulsed laser ablation technique by irradiating a silicon target in an ambient gas (such as argon or helium), which was introduced into the chamber at a constant pressure and gas flow rate. These nanoparticles exhibit a strong red visible luminescence, and the photoluminescence spectrum places the maximum intensity emission at ~690 nm. At room temperature, QDs show a broadband emission spectrum in the approximate range of 400–800 nm under a 325 nm excitation wavelength. The production process and characterization of the nanomaterials (Table 1) are detailed in previous works [24,26]. Briefly, TEM images reveal irregularly shaped nanoparticle particulate aggregates which can be easily disrupted and dispersed by sonication; the Si QDs have a spherical morphology and measure roughly 6–8 nm in diameter. Iron oxide nanoparticles of the α form of hematite (Fe_2_O_3_) were assessed using a high-resolution transmission electron microscope (Philips CM120). The size distribution places them in the range of 10–120 nm (most being between 40 and 60 nm). A Bruker AXS/D8 Advance X-ray diffractometer was used to conduct crystallinity analysis [25]. The characterization of hydrodynamic size and zeta potential was performed in distilled water using a Malvern Zetasizer Nano-ZS instrument (Malvern Instruments, Malvern, UK) with an equilibration time of 1 min at 25 °C using a refractive index of 1.52 for silicon dioxide and 2.93 for hematite. 

### 2.2. Cell Culture

In a preliminary phase, Caco-2 (cat. no. CRL-2102) and HT-29 (cat. No. HTB-38) cell lines purchased from American Type Cell Culture (ATCC, USA) were grown separately in complete medium consisting of Dulbecco’s Modified Eagle Medium (DMEM) supplemented with 10% heat-inactivated FBS and 1% antibiotics. The cultures were maintained in a humid atmosphere at 37 °C with 5% CO_2_ and were routinely subcultured once a week with 0.25% trypsin and 0.53 mM EDTA. For several months, HT-29 cells were treated with methotrexate (MTX) according to the original protocol developed by Lesuffleur et al. [27]. 

Subsequent to the stabilization of HT29-MTX (mucus-secreting) clones, co-cultures were initiated. The cells were seeded on 12-well plates with Transwell^®^ inserts (with polycarbonate membranes, 3 µm pore size, Corning B. V. Life Sciences, USA) at a final density of 100,000 cells per well, regardless of the final seeding ratio (Caco2:HT29-MTX): 10:0, 7:3, 5:5, and 0:10.

### 2.3. Transport Study Design

We exposed Caco-2, HT29-MTX, and co-cultures to non-cytotoxic concentrations (20 μg/mL) of Si QDs and α-Fe_2_O_3_ nanoparticles and to Lucifer Yellow (50 ug/mL). The nanoparticle suspensions were sonicated and dispersed using an ultrasonic processor Hielscher UP50H (Hielscher Ultrasonics GmbH, Germany). Hank’s Balanced Salt Solution (HBSS) was chosen as the transport buffer during the experiment. Particles were quantified by measuring absorbance/fluorescence levels for each (α-Fe_2_O_3_ nanoparticles: absorbance −325/500 nm; Si QDs: wavelength excitation/emission −325/644 nm; wavelength excitation/emission −405/535 nm) using the Flex Station 3 Multireader (Molecular Devices, USA). TEER monitoring was performed using a Millipore® Millicell Electrical Resistance (ERS) system (Millipore, USA). Measurements were performed at three different points of each well, and the final TEER was calculated according to the following formula:

TEER_final_ = (TEER_mean_ [Ω] − TEER_blank_ [Ω]) × A_well_ (1.12 cm^2^) [Ω cm^2^] (3)

### 2.4. Cell Morphology Analysis

At the end of an incubation time of 3 h, Transwell inserts were removed and fixed in 4% paraformaldehyde (PFA) for 24 h before being paraffinized, sectioned (5 μm thick cross-sections), and stained with 1% Alcian Blue-8GX and 0.1% eosin. The procedure was done according to the manufacturer’s kit (Bio-Optica) instructions. Briefly, the sections were placed in distilled water. Alcian Blue pH 2.5 reagent was left to react for 30 min, after which the slides were drained and exposed to sodium tetraborate solution for 10 min. The slides were washed in distilled water before and after treatment with Carmalum reagent (5 min). After a series of dehydration steps using ascending alcohols and xylene, the sections were mounted, and images were captured using an Olympus BX43 (XC30 software). Other cells that were cultured on inserts were fixed with 4% paraformaldehyde for 20 min and permeabilized with 0.1% Triton X-100 and 2% bovine serum albumin for 40 min. F-actin was stained for 30 min with 10 µg/mL phalloidin-FITC (fluorescein isothiocyanate), and the nuclei were counterstained with 2 µg/mL DAPI (4′,6-diamino-2-phenylindole). The slides were examined with an inverted fluorescence microscope Olympus IX71 (Olympus, Tokyo, Japan).

### 2.5. Statistical Analysis

All experiments were performed in triplicate. Data are expressed as the mean value ± standard deviation (SD) of the three independent experiments (n = 3). Statistical differences between samples were analyzed by Student’s *t*-test executed in Microsoft Office software (Excel 2007), and a value of *p* < 0.05 was considered to be statistically significant.

## 3. Results

### 3.1. Monolayer Integrity Assessment 

**Figure 3:**
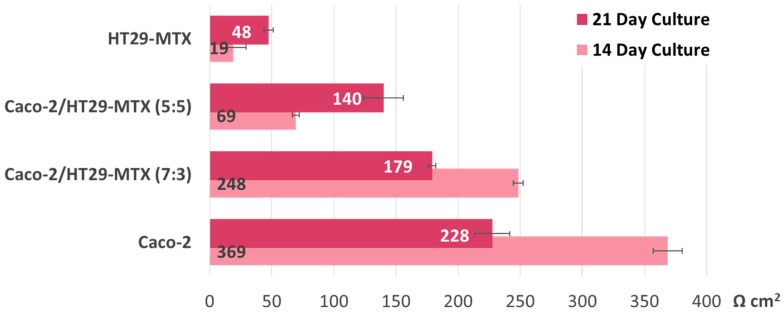
Transepithelial electric resistance (TEER) evolution before initiating the transport study. The TEER values were measured 14 and 21 days after seeding (on Transwell inserts) the Caco-2:HT29-MTX cell cultures in four different ratios: 0:1, 5:5, 7:3, and 1:0. Data are expressed as means ± standard deviation (SD) (n = 3).

The intestinal models tested in the present study recorded similar TEER values to those reported in the literature. After 14 days in culture, the Caco-2 cell line alone produced a very compact monolayer (TEER_μ_ = 369 Ω cm^2^); measurements taken a week after revealed a steep drop to 228 Ω cm^2^, and a similar phenomenon was observed in the case of the Caco-2/HT29-MTX co-cultures seeded at a 7:3 ratio. This would indicate a tissular integrity alteration of around 38%, as seen in Figure 3. The co-cultures initially seeded at equal ratios and goblet cell-like monocultures evolved in a predictable pattern, with steadily increasing TEER values over time. However, the TEER measurements taken 1 week apart for the co-cultures with a higher ratio of Caco-2 (7:3), as well as the Caco-2 monolayers alone, were lower than expected. Nonetheless, after 21 days, the TEER values overall maintained a similar differential rate of increase across models, corresponding to the increasing ratio of Caco-2 initially seeded.

**Figure 4:**
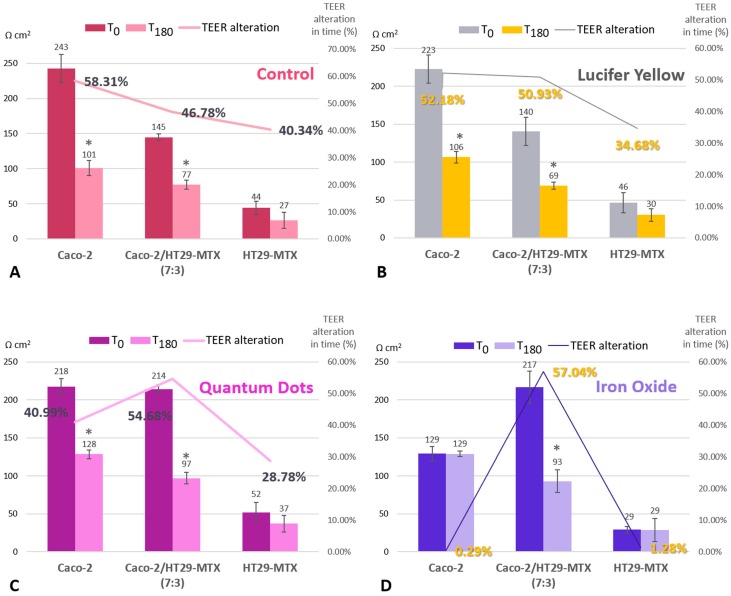
TEER alteration for control cells (**A**), cells incubated with Lucifer Yellow (**B**), with Si QDs (**C**), and with α-Fe_2_O_3_ nanoparticles (**D**). Comparison between three intestinal models (Caco-2, HT29-MTX, and co-culture) before initiating the transport study (T_0_) and after a 3-h exposure to the nanoparticles (T_180_). TEER alteration in time (expressed as a percentage) was calculated in order to compare the difference between the initial values and those measured at 180 min. The calculation was as follows: TEER alteration percent (%) = (T_0_ − T_180_)/T_0_ × 100. Data are expressed as means ± standard deviation (SD) (n = 3). * *p* < 0.05 compared with T_0_.

Regardless of whether the models were exposed to the nanoparticle suspension or not, at the end of the experiment, all groups displayed significantly lower TEER values (see Figure 4), which suggests that other variables at play affect monolayer integrity. Interestingly, when compared with the monocultures, the co-culture models recorded the highest alteration levels, but only if they were exposed to either type of nanoparticle suspension during this time. The 7:3 seeding ratio (Caco-2/HT29-MTX) was selected for further investigations, as it is a better model in terms of approaching the in vivo ratio of enterocytes and mucus-producing goblet cells compared with the other ratios tested. As seen in Figure 3, the Caco-2/HT29-MTX model seeded at a ratio of 7:3 was able to achieve TEER values similar to those cited in the literature [5,28,29], which points to an adequate distribution of tight junctions and confluent cell layer homogeneously covered with mucus.

### 3.2. Comparative Morphological Characterization 

**Figure 5:**
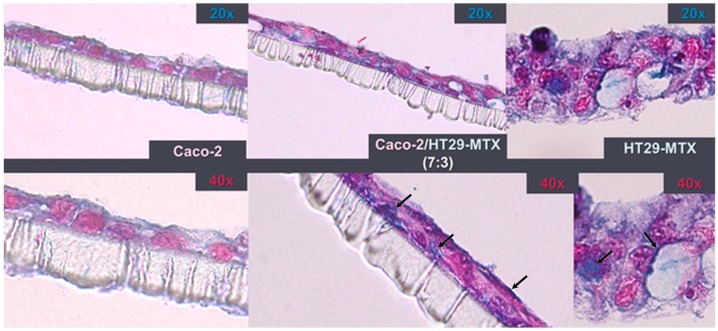
Insert cross-sections after 21 days in culture. Staining with 1% Alcian Blue highlights the production of mucins (black arrows) by HT29-MTX cells. The monolayers were counterstained with 0.1% eosin.

In order to assess whether the HT29-MTX cell line had produced mucus, three inserts were chosen from the control group, each containing a different seeding-ratio developed model, as seen in Figure 5.

Despite the fact that all co-culture models had an identical initial seeding density, Alcian Blue-stained cross-sections show that, after 3 weeks, HT29-MTX monocultures proliferated more, resulting in the formation of a 3D diffused multilayer around the polycarbonate membrane. Compared with the Caco-2 monolayers, the co-culture model exhibited a more intense Alcian Blue staining, which suggests the incorporation of HT29-MTX mucus-producing cells.

**Figure 6:**
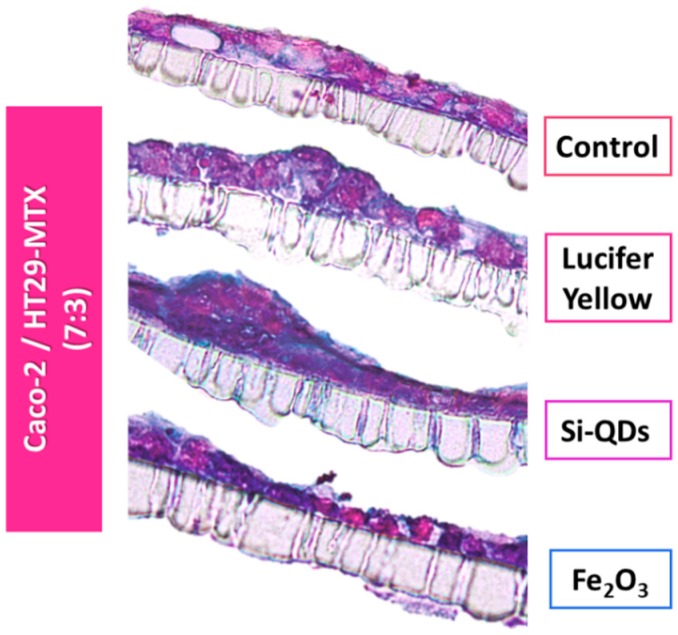
Insert cross-sections of the Caco-2/HT29-MTX intestinal model seeded at a 7:3 ratio following a 3-h exposure to nanoparticles. The cells were stained with 1% Alcian Blue/0.1% eosin in order to detect mucin production. Monolayer disruptions of the experimental groups are visible compared with controls.

**Figure 7:**
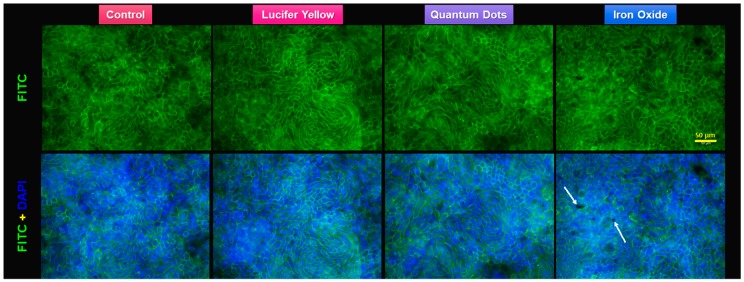
Fluorescence staining of the actin cytoskeleton of Caco-2/HT29-MTX monolayer after the 3-h transport study. F-actin is marked with fluorescein isothiocyanate (FITC)-phalloidin (green). Nuclear counterstain with 4′,6-diamidino-2-phenylindole (DAPI) (blue). Note the aggregates of α-Fe_2_O_3_ nanoparticles (*white arrows*) on the top of the cells.

Alcian Blue staining revealed morphological alterations following the 3-h exposure to Si-QDs or Fe_2_O_3_ nanoparticle suspensions (Figure 6). Comparative characterization of the monolayers after exposure was also conducted using F-actin staining (Figure 7), and the results corroborated the TEER results and the cross-sectional areas stained with Alcian Blue.

### 3.3. Transport Study

**Figure 8:**
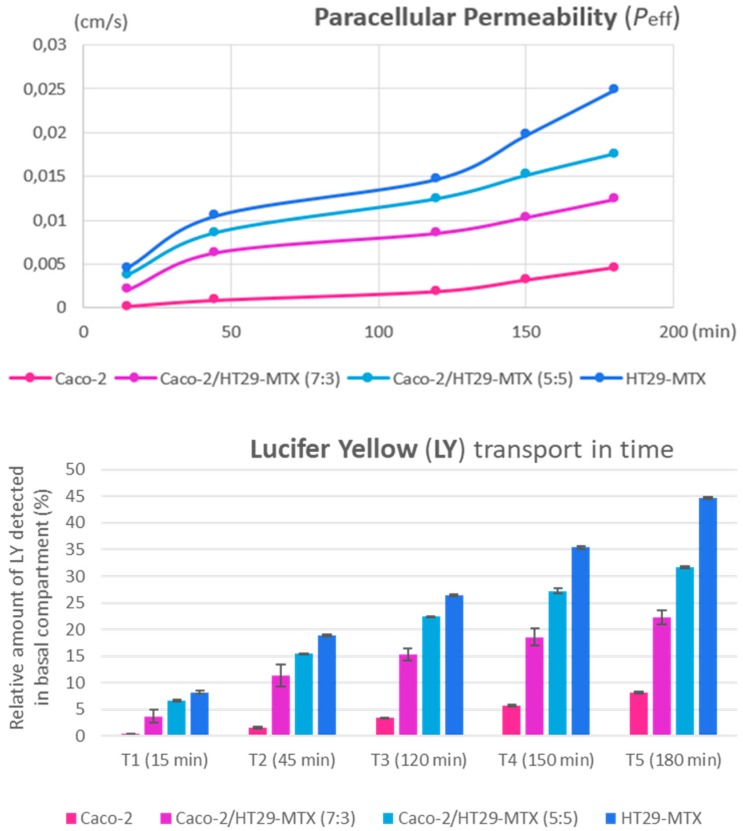
Effective permeability increases across all intestinal in vitro variants (top graph). Lucifer Yellow transport over time (bottom graph) shows the relative amount (in percentages) of the paracellular marker, which was detected at five measurement points during the experiment. Data are calculated as means ± standard deviation (SD) (n = 3).

During the 3-h transport study, neither Si QDs nor Fe_2_O_3_ nanoparticles permeated across the monolayer insert to reach the receiving compartment (data not shown). The paracellular marker Lucifer Yellow passively diffused and reached the basolateral compartment, where it was detected (Figure 8). The Lucifer Yellow transport rate correlates with TEER measurements—the progressive integrity loss of the monolayers and, implicitly, the loosening of tight junction complexes established by Caco-2 cells would result in the increase of effective permeability, even without considering their initial seeding ratio.

## 4. Discussion

Drug permeability coefficients have been historically assessed using simple Caco-2 cultures. This classical intestinal model does present itself with many limitations, one of which is an abnormally high transepithelial electrical resistance (TEER). TEER measurements ensure the consistent monitoring of tissular integrity because it is a highly sensitive, non-invasive technique. TEER values also inversely correlate with paracellular permeability, making it the method of choice for intestinal transport studies [30]. In vivo studies reveal different TEER values between different regions of the human gut; post-confluent Caco-2 monocultures are known to generate signals that vary between 150 and 500 Ω cm^2^, whereas in vivo TEER recordings place the intestinal epithelium in a realistic range of 12–69 Ω cm^2^ [17,30]. This significant difference can be explained by the fact that Caco-2 cells in culture form many more tight junctions. 

When comparing the TEER measurements of co-cultures seeded at different cell ratios (5:5 and 7:3), the data imply convergence in time toward similar transepithelial electrical resistance values (Figure 3). This suggests an internal restructuring process which alters the initial cell type seeding ratio and is congruent with the work of Chen et al. [28], whose study showed, using a Taguchi experimental design, that neither the initial enterocyte-goblet cell ratio nor the cellular density had a significant impact on TEER recordings or the transport of active compounds. Intercellular signaling could account for these results, with both cell lines modulating each other’s growth and proliferation and reaching an equilibrium, regardless of the initial seeding rate. Tumoral cell lines are known to produce and release a series of growth factors which play a crucial role in intestinal development [31]. For example, the expression of epidermal growth factor receptors (EGFRs) in Caco-2 cells is modulated by the substrate on which they are grown, as well as their differentiation stage [32]. Their cellular proliferation is highly affected if they are exposed to vitamin D_3_, which also leads to increases in alkaline phosphatase (ALP) expression [33]. In contrast, insulin-like growth factor 1 (IGF-1) has a positive, dose-dependent effect on Caco-2 proliferation [31]. 

TEER measurements taken 1 week later for the co-cultures with the higher ratio of Caco-2 (7:3) and the Caco-2 monolayers alone were lower than expected (Figure 3). One possible explanation for this is that, unlike HT29-MTX, the Caco-2 cell line has higher requirements in terms of cell culture media, thereby inhibiting cellular expansion in a post-confluent setting. Caco-2 cells grow well on media enriched with 16.5% fetal calf serum (FCS); however, co-culture models must consider the requirements of all the cell lines involved. This study took into account recommendations provided by other research [5] and exposed the cells to a lower concentration of fetal bovine serum (10% FBS). Unlike Caco-2, goblet-like HT29-MTX cells grow well in less strict conditions and require a low amount of glucose; moreover, the cell line is a known producer of lactic acid which can significantly lower the environmental pH. This was observed during handling of the cell line and may account for the lower than expected TEER values of the co-cultures [27].

Previous studies show that many factors, including medium composition and cell culture time, impact TEER values and permeability coefficients of many routinely tested drugs [5,31]. This points to the degradation of the monolayers after a certain time in culture elapses, which results in more permeable intestinal models for compounds that employ the paracellular route. This hypothesis, however, does not account for the higher alteration levels observed for the co-culture models that were part of the experimental groups. This preliminary result suggests that even though the nanoparticle suspensions were dosed such as to not reach cytotoxic levels for either cell line, the co-culture model somehow renders them more vulnerable and more responsive to the materials.

One possible explanation was given by Soto et al. [34], who attributed the cytotoxic effects of some nanoparticulate materials to their inherent tendency to precipitate on top of the cells and form visible aggregates. We noticed a similar phenomenon occurring, prompting further work in order to properly functionalize them and increase their solubility in buffer solutions. Iron oxide nanoparticles are often challenging to work with in this regard—for example, cellular intake of superparamagnetic iron oxide nanoparticle variants (UPSIO NPs) required further functionalization with an oleic acid coating [35]. Even though iron oxide particles are insoluble at physiological temperature and pH, interfacial hydration of anhydrous hematite increases solubility, which enables the cellular uptake of α-Fe_2_O_3_ NPs with the release of ferric ions and generation of hydroperoxyl radicals [25].

QD uptake via the gastrointestinal tract is a new subject that has attracted researchers’ attention due to their biomedical applications, such as in vivo imaging and drug delivery. Degradation of QDs can occur in acidic environments, including the stomach, and can release toxic elements, such as cadmium ions in the case of CdSe-based QDs. Thus, the evaluation of the biodistribution and stability of QDs in the digestive tract is a matter of great interest nowadays. Studies have shown the possibility to modify the surface of particles by different coating approaches in order to achieve their resistance to strong acidic solutions and intestinal permeability [36]. Due to their tendency to aggregate [24], QDs will not easily translocate through the intestinal epithelial barrier [37], as we have seen in this current study. 

Nanoparticle transport across the monolayer was not detected using the quantitative method described in this study. Our working hypothesis is that both types of nanoparticles tested could not diffuse across the monolayer due to their tendency to form aggregates and settle on top of the cells. These nanoparticle aggregates are of a micrometric size and could not be measured by conventional, currently used methods, such as dynamic light scattering (DLS), as this assay requires a properly prepared sample. We measured the hydrodynamic size by DLS (Table 1) of the samples without aggregates, and we obtained values between 250 and 300 nm regardless of the type of particles (quantum dots or iron oxide particles), with the values increasing in the cell culture medium, as previously shown [38]. In addition, the Z-potential of the tested nanoparticles was negative in distilled water, near the value of −30 mV, confirming the negative charge of these particles. We suggest that this Z-potential value did not interfere with the TEER measurements, as we did not observe any difference between the value measured in the Transwell inserts with only PBS (blank) and those only with particles in PBS.

In conclusion, the current study partly achieved its goals concerning the characterization of the in vitro intestinal model Caco-2/HT29-MTX in a transport study setting. Further quantitative testing for the purpose of evaluating nanoparticle toxicity is required, as well as preventing aggregation. 

## Figures and Tables

**Table 1:** Physicochemical characteristics of α-Fe_2_O_3_ and Si quantum dot (QD) nanoparticles as described previously [24,25,26].

Characteristics	α-Fe_2_O_3_ [25]	Si QDs [24,26]
*Form of synthesis*	Powder	Powder
*Method of synthesis*	Laser ablation	Laser ablation
*Purity*	>99%	>99%
*Structure*	α form of Fe_2_O_3_ (hematite)	Crystalline silicon core covered by an amorphous SiO_2_ shell
*Morphology*	Spherical particles	Spherical particles
*Average particle size*	40–60 nm	6–8 nm
*Hydrodynamic size in water*	250–300 nm	250–300 nm
*Z-potential in water*	−30 mV	−27 mV
